# Political priority in the global fight against non–communicable diseases

**DOI:** 10.7189/jogh.02.020403

**Published:** 2012-12

**Authors:** Anthony Maher, Devi Sridhar

**Affiliations:** 1 Faculty of Law, McGill University, Montreal, Quebec, Canada; 2 Global Public Health Unit, Edinburgh University, Edinburgh, Scotland, and Blavatnik School of Government, Oxford University, Oxford, England, UK

## Abstract

**Background:**

The prevalence of non–communicable diseases (NCDs) – such as cancer, diabetes, cardiovascular disease, and chronic respiratory diseases – is surging globally. Yet despite the availability of cost–effective interventions, NCDs receive less than 3% of annual development assistance for health to low and middle income countries. The top donors in global health – including the Bill and Melinda Gates Foundation, the US Government, and the World Bank – together commit less than 2% of their budgets to the prevention and control of NCDs. Why is there such meagre funding on the table for the prevention and control of NCDs? Why has a global plan of action aimed at halting the spread of NCDs been so difficult to achieve?

**Methods:**

This paper aims to tackle these two interrelated questions by analysing NCDs through the lens of Jeremy Shiffman’s 2009 political priority framework. We define global political priority as ‘the degree to which international and national political leaders actively give attention to an issue, and back up that attention with the provision of financial, technical, and human resources that are commensurate with the severity of the issue’. Grounded in social constructionism, this framework critically examines the relationship between agenda setting and ‘objective’ factors in global health, such as the existence of cost–effective interventions and a high mortality burden. From a methodological perspective, this paper fits within the category of discipline configurative case study.

**Results:**

We support Shiffman’s claim that strategic communication – or ideas in the form of issue portrayals – ought to be a core activity of global health policy communities. But issue portrayals must be the products of a robust and inclusive debate. To this end, we also consider it essential to recognise that issue portrayals reach political leaders through a vast array of channels. Raising the political priority of NCDs means engaging with the diverse ways in which actors express concern for the global proliferation of these diseases.

**Conclusion:**

Ultimately, our political interactions amount to struggles for influence, and determining which issues to champion in the midst of these struggles – and which to disregard – is informed by subjectively held notions of the right, the good, and the just. Indeed, the very act of choosing which issues to prioritise in our daily lives forces us to evaluate our values and aspirations as individual agents against the shared values that structure the societies in which we live.

The prevalence of non–communicable diseases – such as cancer, diabetes, cardiovascular disease, and chronic respiratory diseases – is surging globally. In 2004 deaths due to NCDs accounted for three out of five deaths worldwide, with 80% of these deaths occurring in low– and middle–income countries [1]. What is more, deaths due to NCDs are predicted to increase by 15% worldwide between 2010 and 2020 [2].

NCDs are no longer just the scourge of the rich. As the World Health Organization (WHO) recently observed: “*NCDs and poverty create a vicious cycle whereby poverty exposes people to behavioural risk factors for NCDs and, in turn, the resulting NCDs may become an important driver to the downward spiral that leads families towards poverty*” [3]. A recent World Bank study in India found that treatment costs for an individual with diabetes typically consume between 15 to 25% of household earnings [4]. Where families lack access to affordable health care – a reality that is especially commonplace in low– and middle–income countries – they tend to forego care or fall into financial hardship; in both cases, the poor end up suffering the worst [5].

Moreover, the main risk factors for NCDs are perpetuated through social norms and practices. According to the WHO, these risk factors include tobacco use and exposure to second–hand smoke, unhealthy diet, physical inactivity, and harmful use of alcohol [2]. The impacts of these factors are not immediately detectable (as in the case of an infectious virus), but evolve over the course of one’s lifetime. NCDs can thus be described as ‘invisible’ diseases: their long–term nature makes it such that sufferers often go unnoticed.

NCDs are thus rooted in the social determinants of health and cannot be stopped through individual action alone. By way of example, current global marketing activities are driving the transition towards diets that are high in sugar and saturated fat, thus increasing the risk of developing one or more NCDs [6]. Research has also demonstrated strong links between increased tobacco consumption, free trade, and foreign direct investment. For instance, in the 1980s bilateral trade agreements signed between the US and several countries in Asia resulted in a spike in demand for tobacco products, especially in the poorest Asian countries [7].

Recognising the need for a collective response, the WHO has responded with a shortlist of ‘best buy’ policy interventions to prevent and treat NCDs. These policies include tax increases to curb tobacco use; restrictions on the marketing of alcohol; replacement of trans fats to promote healthier diets; hepatitis B immunisation to prevent liver cancer; and multi–drug therapy to prevent heart attacks and strokes [8]. Most significantly, these ‘best buys’ can be implemented at relatively low cost, ranging from US$ 1.50–2.00 per head in low–middle income countries to US$ 3 per head in upper–middle income countries [8]. The costs of inaction are much greater: the WHO estimates that each 10% rise in NCDs is associated with 0.5% lower rates of annual economic growth [3]. Thus, in 2011 the World Economic Forum ranked NCDs among the major global threats to economic development [9].

Yet despite the availability of cost–effective interventions, NCDs receive less than 3% of annual development assistance for health to low and middle income countries [10]. The top donors in global health – including the Bill and Melinda Gates Foundation, the US Government, and the World Bank – commit less than 2% of their budgets to the prevention and control of NCDs [11]. NCDs cause the highest burden of disease across the world, and yet the global response to this reality has been woefully inadequate.

Why is there such meagre funding on the table for the prevention and control of NCDs? Why has a global plan of action aimed at halting the spread of NCDs been so difficult to achieve? This paper aims to tackle these two interrelated questions by analysing NCDs through the lens of Jeremy Shiffman’s (2009) political priority framework [12]. We define global political priority as “*the degree to which international and national political leaders actively give attention to an issue, and back up that attention with the provision of financial, technical, and human resources that are commensurate with the severity of the issue*” [13]. Grounded in social constructionism, this framework critically examines the relationship between agenda setting and technical factors in global health, such as the existence of cost–effective interventions and a high mortality burden. Shiffman calls into question the tendency on the part of many global health advocates to treat indicators of the burden of disease as self–evident. To this end, Shiffman argues that strategic communication surrounding the causes, effects, and implications of disease ought to be a central task of health advocates. Following this logic, we explore the ways in which the policy community surrounding NCDs – or the network of individuals and organisations concerned with the issue – have come to understand and portray the issue’s importance. In this manner, we explain the neglect of NCDs on the global stage in terms of a lack of strategic communication.

We begin by outlining the theoretical approach and methodology to be followed throughout the paper. Next, we explore the various ideas and framing mechanisms that have been used to portray NCDs. Finally, we seek to address two weaknesses in the Shiffman framework. We conclude by reconciling the Shiffman framework’s focus on strategic communication with the claim, advanced by several global health experts, that well–financed corporate and private agendas currently act to undermine the pursuit of health for all.

## SOCIAL CONSTRUCTIONISM

This paper follows in the social constructionist tradition, whose most basic tenet holds that ‘[our] socially shared interpretations mediate and form our perceptions of reality’ [12]. While the social construction of reality is well established in social scientific research [14-16], this approach has been applied in only a handful of instances in the field of public health [17,18]. This paper thus aims to show the rich insights to be gained from applying a social constructionist approach to the study of human disease.

In order to attract attention for an issue, we argue that actors must engage in ‘strategic social construction’. This is defined as the process whereby actors conduct means–ends calculations with a view to changing other actors’ utility function in ways that reflect new normative commitments [19]. Moreover,we operate from the assumption that a desire on the part of a group of actors to transform ideas into norms can be meaningfully translated into an effective plan of action; in other words, that ‘we can think about the strategic activity of actors in an intersubjectively structured political universe’ [20].

Consequently, we use Shiffman’s (2009) political priority framework as a tool for understanding the process of translating grievances into norms that demand action. Shiffman identifies three variables that are fundamental to raising the priority of a given issue area: (1) ideas, (2) institutions, and (3) policy communities. The framework to form the basis of the present analysis is a condensed version of an earlier framework proposed by Shiffman and Smith (2007). We use the 2009 version of the framework as it makes explicit its critique of materialist approaches that explain health priority–setting in terms of ‘objective’ indicators.

From a methodological perspective, this paper fits within the category of discipline configurative case study [21]. This type of study uses established theories to explain a case, whether for the purpose of highlighting important historical developments, improving pedagogy, or drawing attention to the need for new theory in neglected areas. One limitation of this approach is the temptation to make predictions about future events on the basis of theories that ‘lack clarity and internal consistency’ [21]. To date, the Shiffman framework has been applied to a limited number of cases, including maternal and child health, neonatal health, and oral health [13,22-24]. Beyond these cases, the framework’s theoretical implications remain unspecified. We endeavour to surmount this limitation by clarifying and refining the framework. A second limitation of this paper is its lack of interview data. However, wherever possible we incorporate primary source material, including direct statements from actors in the public sector, private industry, and civil society. Third, this paper does not focus on the institutional factors that have impeded the generation of priority for NCDs. While we acknowledge that well–financed institutions are crucial in terms of giving ‘teeth’ to an issue, this paper aims to refine the framework’s theoretical assumptions. If incorporated into future analyses, these refinements can be used to situate the role of institutions in the priority generation process.

## THE USE OF IDEAS IN NCD ADVOCACY

What ideas have been used to portray NCDs? What ideas have been ignored? This will be accomplished by analysing these ideas from three vantage points: issue framing, issue characteristics, and implementation. Framing refers to ‘conscious strategic efforts by groups of people to fashion shared understandings of the world and of themselves that legitimate and motivate collective action’ [25]. It follows from this definition that issues in global health do not automatically designate themselves as priority issues, but rather, that issues are selectively and consciously advanced by organised groups of people. Crucially, however, the frames that condition these strategic decisions often go unnoticed. As such, ‘[we] do not see the frame directly, but infer its presence by its characteristic expressions and language. Each frame gives the advantage to certain ways of talking and thinking, while placing “other out of the picture”’ [26].

Why, then, do certain frames resonate with political leaders and the public at large and subsequently compel action, while others do not? Two characteristics that can be used to explain this variance in the efficacy of frames are ‘credibility’ and ‘salience’ [27]. Credibility refers to ‘how truthful people perceive the frame to be’, whereas salience refers to ‘how central [the frame] is to their lives’ [12]. To zero in on how credibility and salience are portrayed, Shiffman (2009) identifies two types of claims generally used by activists in global health: problem claims, surrounding severity and neglect of their issue; and solution claims, surrounding a given problem’s tractability and the benefits that would ensue from addressing it. An example of a problem claim from the literature on NCDs is as follows: ‘An urgent and collective response is required because no country alone can address a threat of this magnitude’ [5]. Conversely, in the aim of drawing attention to the need for concerted action on NCDs, other advocates have put forth the following solutions claim: ‘The evidence is unequivocal: major and rapid health and economic gains are possible with only modest investments in prevention and control of chronic diseases’ [28].

The purpose of these claims is to convince others to ‘buy into’ the interpretations that they advance. For instance, the notion of ‘magnitude’ in the aforementioned problem claim is invoked in order to make a normative judgment. In this particular claim, magnitude is linked with the notion of a ‘collective response’, or the capacity of human beings to affect meaningful change. As such, the intended effect of this claim is to exclude interpretations that reduce the proliferation of NCDs to individual responsibility by framing the issue in terms of unrealised potential for collective action.

Credible claims, therefore, are ones that align with previously held frames. But what role has framing played in contributing to the lack of priority for NCDs? What frames have been used to portray this issue, and how can we assess the effectiveness of these frames?

First, the issue of credibility has been dominated by calls to improve surveillance of these diseases, particularly in the developing world. The WHO’s 2008–2013 Global Strategy for the Prevention and Control of Noncommunicable Diseases repeatedly identifies the elaboration of ‘reliable population–based mortality statistics and standardized data’ as a key strategic objective [29]. Indeed, for one well–versed in quantitative methods, the availability of credible facts demonstrating the effects of NCDs may be enough to motivate action. In response to enduring confusion about the causes of NCDs such as lack of personal control, the lead author of one of the papers in the first *Lancet* series on chronic diseases thus remarked: ‘I thought we got rid of these myths. But they keep coming back’ [30].

However, for others in government and in industry, mere reliance on statistics may prove unconvincing. Indeed, a wide range of factors has served to reinforce the perception that NCDs are unworthy of attention. For instance, the very label of these diseases is a case study in poor branding: “anything that begins with ‘non’ may be considered a ‘non–issue’ or a ‘non–starter’” [31]. Moreover, it fails to convey the crucial point that NCDs are indeed communicable: not just through infectious modes of transmission, but also through social norms and practices. In China, for instance, 59% of Chinese men smoke, compared to only 4% of women [32]. In the Chinese context, smoking – a key risk factor for NCDs – is therefore interwoven with gender roles and perceptions of social status. A label that at first glance excludes social processes as forms of disease communication thus represents a major impediment to the generation of priority.

The lack of a ‘human face’ to portrayals of NCDs represents a second problem. In contrast to a disease such as polio, where the victim is immediately recognisable by virtue or his or her physical appearance, sufferers of diseases such as diabetes often go unnoticed. To suggest that evidence alone can compel action is to ignore the role of emotion and affect in shaping human reactions to external events [33]. Yet NCDs have not been portrayed through the use of images and media clips that depict actual human sufferers [34]. This serves to dehumanise the issue, limiting its emotional appeal, and ultimately, its salience.

These examples suggest that credibility, understood in terms of technical evidence, has dominated the debate surrounding NCDs. Crucially, this has happened at the expense of salience. The role of framing in conveying the implications of mortality statistics is often misunderstood (as evidenced by *The Lancet* lead author’s above comment), or ignored entirely. The myths that, for some, evidently contradict convincing scientific evidence can seem irrelevant for others whose frames of reference do not consider such myths to be worthy of attention. As a result, intentionally shaping social norms and practices involves much more than outwardly projecting facts and figures and hoping that they ‘stick’. In the absence of context–sensitive communication strategies, claims surrounding the severity or tractability of NCDs may never make it off the page.

How, then, do certain the technical aspects of NCDs interact with ideas in the priority generation process? We focus on three variables that mediate this relationship: (1) ‘causes [that] can be assigned to the deliberate actions of identifiable individuals’; (2) ‘issues involving bodily harm to vulnerable individuals, especially when there is a short and clear causal chain assigning responsibility’; and (3) ‘issues involving legal equality of opportunity’ [35].

In terms of the first and second factors, the fact that NCDs are caused by several risk factors and over the course of a long period of time makes it difficult to attribute their causes to the deliberate actions of identifiable individuals. Ultimately, the perceived uncertainty about NCDs, and about many public health problems in general, is a function of causality [17]. As a result, the lack of attention for NCDs is at least partly attributable to a failure to engage with ideas of causality.

Similarly, the third factor identified above – legal equality of opportunity – represents another obstacle to the generation of priority for NCDs. To date, the WHO has not used its treaty–making power in order to articulate and enforce legally binding regulations surrounding NCDs. However, the WHO did exercise this power in 2005 in order to establish the Framework Convention on Tobacco Control (FCTC). The fact that cigarettes contain carcinogenic tar and other harmful agents – a material component of tobacco – provided sufficient rationale for the establishment of a legally binding treaty designed to curb tobacco use. The FCTC demonstrates that certain interpretations of the causes of disease are so widely shared that it is feasible to control them through the use of legal instruments. However, there has been little headway made towards a Framework Convention on Alcohol Control [36]. From a legal standpoint, the lack of a short causal chain for the full range of risk factors for NCDs thus represents a core challenge for global health advocates.

Another example of the interplay between technical factors and ideas is captured by the role of consumer insights in food and beverage production. Private industry has actively called for further research ‘to gain a better understanding of the biology of sweeteners in human sensory systems’ [37]. This argument holds that in order to meaningfully halt the spread of NCDs, consumer taste preferences must be taken into account. PepsiCo has thus committed to remove 10 210 tonnes of salt from its products sold in the US by 2015 – and this, without compromising flavour [37].

However, it remains the case that these taste preferences are always mediated. For example, PepsiCo’s commitment to maintaining a wide range of product offerings reproduces ideas about the nature of consumption in a global marketplace. The very suggestion of responding implies that humans will continue to demand as many food and beverage options as possible in accordance with a free market mentality. Yet food is not seen as a symbol of free–market choice in all social contexts. Simply labelling certain foods ‘bad’ in excess quantities assumes a common interpretation of the role these foods play in the lives of those who consume them. In developing world contexts, for instance, access to a variety of food options may be much more limited than in Western societies. In these contexts, acommoditised understanding of food may play little to no role in shaping local dynamics. A major gap thus lies in understanding the ways in which social meanings interact with potentially harmful foodstuffs, particularly in the informal sector and in home food preparation [37,38]. In accordance with the Shiffman framework, disaggregating the different views attached to technical factors such as food, and examining the ideas and value systems upon which they are based, is crucial to devising effective strategies to elevate the importance of NCD prevention and control.

One could therefore ask: Is it necessary to have an elaborate base of evidence justifying a proposed intervention in order to generate support for that intervention? As previously mentioned, such a focus on expanding the evidence base is frequently invoked by public health experts. As a further example, one of the key problems identified at the United Nations High–Level Meeting on the Prevention and Control of Non–communicable Diseases held in September 2011 was the lack of ‘a proper evaluation on the differences between community and targeted initiatives’ with respect to minimum age regulations for youth [34].

Of course, we do not dispute that the availability of rigorous evidence is crucial to achieving better health outcomes. At the same time, the availability of evidence is merely one component that political leaders consider when deciding which issues to prioritise. Bull and Bauman (2011) echo this argument in reference to one of the key risk factors for NCDs, physical activity, calling ‘inaccurate’ the perception that we do not have sufficient evidence to act. Instead, these authors assert that ‘[much] better use of well–planned, coherent communication strategies are needed’ [39].

In this vein, we argue that a useful way of understanding the generation of political priority for a given issue is to reflect on the ideas attached to proposed interventions related to that issue. If one accepts that the availability of evidence is one among many factors that motivate policy–making, understanding the underlying reasons that inspire the actions of policy–makers is crucial. In the case of NCDs, this would suggest that the availability of cost–effective interventions ought not to be ignored in the process of devising effective issue portrayals.

As an example in this regard, one can consider the many connotations of the term ‘epidemic’. One of the most salient debates in the lead–up to the UN High–Level Meeting on NCDs centred on the implications of labelling the spread of NCDs an ‘epidemic’. Applying this label to NCDs could allow countries to invoke flexibilities in World Trade Organization rules that allow drug manufacturers to make generic versions of patented drugs [40]. These flexibilities find their origin in a provision in the Doha Declaration on the TRIPS Agreement and Public Health, which holds that ‘public health crises, including those relating to HIV/AIDS, tuberculosis, malaria and other epidemics, can represent a national emergency or other circumstances of extreme urgency’ [41]. In the end, the draft political declaration agreed by World Health Organization Member States referred to NCDs as a ‘challenge of epidemic proportions’ [42].

For many in public health, calling the global spread of NCDs an epidemic reflects the very real need for urgent action. This interpretation appeals to language traditionally used to refer to other diseases such as HIV/AIDS, on the basis that framing NCDs in a similar way will attract high levels of support. From this perspective, one could argue that to water down the impacts of NCDs by referring to them in any other way would be the equivalent of willfully ignoring sound evidence.

However, for others in government and in the business community, the term ‘epidemic’ conjures up scenarios of stifled innovation and, its corollary, ineffective pharmaceutical products. For example, the director of the Office of Global Affairs at the US Department of Health and Human Services justified US opposition to eliminating all patent protections on drugs that treat NCDs as follows: “[Doing away with patent protections], *to our minds, was not the way you get a stream of ongoing research and development and the new and improved drugs that we continue to need*” [40].

In terms of reconciling these conflicting positions, the Shiffman framework offers the following insight: Policies that may seem entirely rational in one social context may seem irrational in another. Just because an intervention *can* be understood by an audience does not mean that it *will* be understood in the way in which one intends. Thus, a strictly public health perspective on NCDs that ignores the broader economic implications of proposed interventions spread will have little appeal amongst these actors.

What is more, this particular understanding of the relationship between health and the economy is informed by socially constructed – or, in this case, free–market capitalist – ideas about the global economic order. This demonstrates that decisions on the part of political leaders regarding whether or not to implement policies aimed at the prevention of disease are inseparable from broader questions of ideology. Policies related to health are much more than just instrumental means of achieving a result of maximum ‘utility’, but are inspired by value systems that cannot be explained by rational calculations alone [19]. As a consequence, raising the political priority of a given issue is contingent upon engagement with the underlying rationales of policy proposals related to that issue. The full range of interpretations associated with proposed interventions must therefore be taken into account in the process of devising effective portrayals of NCDs and their effects.

## DEFINING THE NCD POLICY COMMUNITY

In this final section we reflect on what constitutes the policy community surrounding NCDs. We address this question by proposing two specific refinements to the Shiffman framework: first, to broaden the definition of policy community to include private industry; and second, to reconceptualise the structural determinants of NCDs in terms of ideas.

Shiffman defines policy communities as ‘networks of individuals (including researchers, advocates, policy–makers and technical officials) and organizations (including governments, non–governmental organizations, United Nations agencies, foundations and donor agencies) that share a concern for a particular issue’ [12]. However, Shiffman does not specify what he means by ‘sharing concern’ for an issue. From an analytical point of view, this generates significant uncertainty. The lack of clarity surrounding the notion of concern can lead one to focus too narrowly on the community of actors who actively proclaim to be in support of an issue, while failing to incorporate the influence of those ‘outside’ this community. To this end, “[an] *analytic approach that offers policy community actors as the central creators and disseminators of ideational messages misses other possible sources of ideas that may prove persuasive in motivating action*” [24].

The desire on the part of many actors in the private sector to align profit objectives with broader social goals also represents a way of expressing concern, and one that has proven highly influential. As such, we seek to amend Shiffman’s definition of policy community by explicitly including the private sector. We argue that an issue is concerning to an individual or set of actors when it is of interest, or of importance, to that individual or set of actors. This definition allows for concern to be expressed in many different ways, and not just through traditional methods, such as protest or lobbying. Under this definition, strategic communication is still essential to raising the priority of a given issue area, but the source of such communication is expanded to include a wider range of actors. In this manner, actors that have traditionally been excluded from the policy community and portrayed as forces to be resisted – such as multinational corporations in global health – are redefined as agents of change.

The funding trends are clear: private donors are increasingly driving the global health agenda. Furthermore, the role of private corporations in shaping public perceptions about the risk factors for disease extends well beyond the realm of aid for health. In the domain of advertising, for instance, 11 multinational companies – including such well–known companies as General Mills, Nestlé, Mars, and PepsiCo – account for approximately 80% of global advertising spending in the food and beverage industry [37].

Much less clear, however, is the question of how to respond to these trends. One notable response is the adapted political process model [30]. Among other factors, this model identifies vested corporate interests as a key obstacle to meaningful action on NCDs, claiming that such interests are often subversive, or, to use their words, ‘diabolical’ in nature [30]. These authors are also critical of the Shiffman framework. They argue that it “[views] *politics as a market, where the ultimate political outcomes are determined by a collision of forces involving people, interests groups, and ideas*”. To those who would seek to advance the Shiffman framework, they present the following challenge: “[does] *it help to tell someone that their ideas about how to control diseases have not been influential, so they should come up with better ones*?” [30].

In our view this challenge is misguided in several respects. First, the act of strategic communication cannot be reduced to ‘good’ vs ‘bad’ ideas. This misses the crucial point that our frames paint certain ideas as unworthy of attention from our very first encounter with them. This does not mean that such ideas are intrinsically ‘bad’, but that they may not resonate with our target audience. In order to get past frames – the ‘gatekeepers’ of ideas – the ideas that we employ must be salient in the lives of those whom we intend to influence.

Second, this challenge overlooks the constructed nature of interests. The authors of the political process model do indeed acknowledge the need to identify corporate interests, concluding with the following recommendation: “*A challenge for global health is to identify these interests and bring them to the light of day, holding them to standards of transparency and public accountability*” [11]. But this argument fails to recognise that ‘they’ are also ‘us’. ‘They’ – in this case, private corporations – respond to, influence, and are legitimated by, the ideas that ‘we’ hold. Their very existence is contingent upon consent. To suggest that corporate interests must be resisted due to their ‘diabolical’ nature is to depict these interests as irreconcilable with the interests of other (implicitly more benevolent) actors who operate within a given issue area. In short, it is to take the intentions, interests, and attitudes of these actors as granted. But more than that, it is to incite feelings of animosity towards private corporations whose interests may be much more complex than such feelings may lead one to believe. As a result, the adapted political process model may lead to oversimplifications that focus on dominant interpretations at the expense of alternative ones, and taken to the extreme, portray certain issues as polarised to the point of being beyond the reach of mutual dialogue.

This is not to suggest that corporations always act in the best interests of human health; indeed, history is replete with examples of corporate entities using ethically questionable marketing tactics and failing to internalise the environmental and social costs of their operations. In this regard, one can consider the ‘Keep America Beautiful’ campaign in the United States that was aimed at reducing street litter and promoting environmental awareness. Funded by the tobacco conglomerate Philip Morris, this campaign targeted every kind of trash except tobacco waste, despite the fact that tobacco is estimated to make up 25% of all litter on US streets [30]. This case clearly contradicts well–established evidence about the negative health effects of smoking.

Nevertheless, rather than categorically excluding corporations from the policy community, we contend that a more valuable approach is to focus on the contested ideas emerging within the modern–day economic system – even if these ideas are more incremental than alarmist in their assessment of the system’s shortcomings. The concept of corporate social responsibility is most illustrative of this point. This concept holds that ‘business models should marry performance and profitability with the deliberate purpose or goal of contributing to the solution of relevant social and environmental challenges’ [37]. Under the leadership of CEO Indra Nooyi, PepsiCo has thus adopted the phrase ‘Performance with Purpose’ to guide its operations. Similarly, the US Secretary of Health Kathleen Sibelius recently remarked: “*Healthy offering and healthy profits are not mutually exclusive*” [34].

In this regard, the success of the business community in attracting the support of US government and other high–level officials is due in no small part to the appeal of its overarching ethos: to “[provide] *consumers with the tools they need to maintain a healthy lifestyle*” [37]. This consumer–centric focus is influential in two key respects. First of all, many Western states have reduced their foreign aid budgets in the wake of the 2008–2009 global financial crisis, thus limiting the funds available for global health initiatives. Second, governments must invariably confront other social and economic problems, including financial instability, terrorism, and climate change. While many of these problems can be addressed synergistically, the fact remains that global health advocates often compete with these issues for attention. As a result, strategies for improving health outcomes that are consumer–focused and that take the bulk of responsibility off the shoulders of government are, in many cases, more likely to gain traction.

To be clear, we do not intend to argue in favour of greater private sector involvement in halting the spread of NCDs. We simply mean to highlight the power of the ideas employed by the private sector, and to suggest that any supposedly balanced analysis of NCDs must take them into account.

Finally, it is worthwhile to address relationship of social constructionism to notions of power. One major criticism of this approach holds that that simply identifying ideational constructs fails to address the powerful inequalities that restrict the ability of individuals to communicate ideas in the first place [43]. It follows that “[too] *much emphasis on the message can draw our attention away from the carriers of frames and the complicated and uneven playing fields on which they compete*” [26]. Similarly, engaging with power relations addresses the criticism that constructivists shy away from seemingly ‘evil’ norms and ideas [44]. Indeed, the dominant economic paradigm at present is one of free markets, trade liberalisation, and consumer choice. Particularly within the alternative globalisation movement, these free market forces are often considered predatory to the point of being evil – and it is clear that the private sector is a key driver of these forces [45].

A concrete example of an attempt to reorient the perception of the private sector as a structural force to be resisted is the Pan American Health Organization Forum for Action on Chronic Disease, also known as the Partners Forum. It has engaged the private sector by including business representatives in a reworked version of the CARMEN network, which is comprised of 32 countries in the region of the Americas that are committed to the prevention and control of NCDs and their risk factors. A key element of this Forum is the establishment of a ‘clear definition on who the members should be, criteria for inclusion, admission, and rules for removal’ [46]. In short, the Partners Forum represents a significant step forward in terms of reconciling the many ways in which that global health advocates – including private companies – express concern, but for reasons of fear, pride, or otherwise, fail to operationalise in the form of partnerships.

Moreover, it is clear from this example that the act of strategically communicating ideas about the social determinants of health is much more than just a one–way transfer of information. That some in global health see the private sector as a structural force to be resisted is the result of blaming a subset of actors that is not solely responsible for producing the current situation. Power is not something that actors automatically possess, the crucial point being that movements develop *within* communities, and not from the exploits of individual actors working in isolation [26]. In this regard, social structures ought not be understood not as monolithic forces to be resisted [47]. On the contrary, the process of advancing new normative commitments ought to be understood as a process of socialisation, through which boundaries between contested and shared ideas are debated, articulated, and redefined.

## CONCLUSION

In conclusion, through the lens of Shiffman’s political priority framework, we have sought to shed light on the factors that have relegated NCDs to the bottom of the agendas of governments and donors in global health. Our objective has not been to dictate what global health advocates should do in order to raise the priority of NCDs. Instead, we have attempted to elucidate the perceptions that have led to NCDs being ignored in the corridors of power. By deconstructing the attitudes, interests, and motivations of relevant national, international, and transnational actors in global health, we have sought to identify the ways in which these perceptions have been reproduced. This, in turn, enables advocates to communicate the causes and potential impacts of NCDs in a way that is sensitive to existing points of view.

We support Shiffman’s claim that strategic communication – or ideas in the form of issue portrayals – ought to be a core activity of global health policy communities. But issue portrayals must be the products of a robust and inclusive debate. To this end, we also consider it essential to recognise that issue portrayals reach political leaders through a vast array of channels. This means acknowledging the role of actors, such as private entities whose intentions may not at first glance appear to be shared with the traditional members of a policy community, such as researchers, physicians, and NGOs. Raising the political priority of NCDs means engaging with the diverse ways in which actors express concern for the global proliferation of these diseases. In the case of the private sector, this means recognising that companies often choose to pursue both economic and social goals in an integrated manner. As we have argued, portrayals of NCDs have been hampered by dissonance between the ideas espoused by actors in the public, private, and civil society sectors; a prominent example of this being the polarisation of the debate over whether to label the global spread of NCDs an epidemic. Promoting dialogue between these actors is a crucial first step in terms of devising communication strategies that are likely to resonate and compel action.

More broadly, we have endeavoured to show the value of social constructionism as an approach to the study of social and political change. The following insight is most instructive: “*Persuasion is the process by which agent action becomes social structure, ideas become norms, and the subjective becomes the intersubjective*” [19]. Strategic portrayals of ideas thus constitute the practical equivalent of translating agent action into structure.

Indeed, this analysis opens up several avenues for further research. First, how have the frames used to portray NCDs varied over time? What criteria can we use to study the long–term success of issue portrayals, and how can we measure salience in the long–term? Second, this analysis also demonstrates the importance of studying unsuccessful attempts at attracting high–level attention for a given cause. In what ways have other neglected health problems been understood and portrayed? What commonalities do these portrayals share with those used by advocates for NCDs?

Ultimately, our political interactions amount to struggles for influence, and determining which issues to champion in the midst of these struggles – and which to disregard – is informed by subjectively held notions of the right, the good, and the just. Indeed, the very act of choosing which issues to prioritise in our daily lives forces us to evaluate our values and aspirations as individual agents against the shared values that structure the societies in which we live.
